# Concept for Recycling Waste Biomass from the Sugar Industry for Chemical and Biotechnological Purposes

**DOI:** 10.3390/molecules22091544

**Published:** 2017-09-13

**Authors:** Magdalena Modelska, Joanna Berlowska, Dorota Kregiel, Weronika Cieciura, Hubert Antolak, Jolanta Tomaszewska, Michał Binczarski, Elzbieta Szubiakiewicz, Izabela A. Witonska

**Affiliations:** 1 Institute of General and Ecological Chemistry, Faculty of Chemistry, Lodz University of Technology, Zeromskiego 116, 90-924 Lodz, Poland; magdalena.modelska@dokt.p.lodz.pl (M.M.); trukan.trukan@gmail.com (J.T.); michalbinczarski@gmail.com (M.B.); elzbieta.szubiakiewicz@p.lodz.pl (E.S.); 2 Institute of Fermentation Technology and Microbiology, Faculty of Food Science and Biotechnology, Lodz University of Technology, Wolczanska 171/173, 90-924 Lodz, Poland; dorota.kregiel@p.lodz.pl (D.K.); weronikacieciura@wp.pl (W.C.); hubert.antolak@gmail.com (H.A.)

**Keywords:** acid hydrolysis, biomass, furfural, biotechnological processes, lactic acid

## Abstract

The objective of this study was to develop a method for the thermally-assisted acidic hydrolysis of waste biomass from the sugar industry (sugar beet pulp and leaves) for chemical and biotechnological purposes. The distillates, containing furfural, can be catalytically reduced directly into furfurayl alcohol or tetrahydrofurfuryl alcohol. The sugars present in the hydrolysates can be converted by lactic bacteria into lactic acid, which, by catalytic reduction, leads to propylene glycol. The sugars may also be utilized by microorganisms in the process of cell proliferation, and the biomass obtained used as a protein supplement in animal feed. Our study also considered the effects of the mode and length of preservation (fresh, ensilage, and drying) on the yields of furfural and monosaccharides. The yield of furfural in the distillates was measured using gas chromatography with flame ionization detector (GC-FID). The content of monosaccharides in the hydrolysates was measured spectrophotometrically using enzymatic kits. Biomass preserved under all tested conditions produced high yields of furfural, comparable to those for fresh material. Long-term storage of ensiled waste biomass did not result in loss of furfural productivity. However, there were significant reductions in the amounts of monosaccharides in the hydrolysates.

## 1. Introduction

Sugar beet is one of the most important agricultural crops in Europe. Each year, approximately 200 million tons of sugar beet is produced in Europe, around two thirds of world production. The main product of processing sugar beet is sugar (saccharose) [1]. However, after the extraction of saccharose, approximately 68 million tons of wet sugar beet pulp (SBP) or 17 million tons of dried biomass is also generated worldwide [2]. This agro-waste consists of polysaccharides (22–24 wt. % cellulose and 30 wt. % hemicelluloses) and pectin (15–25 wt. %), with small amounts of fat (1.4 wt. %), protein (10.3 wt. %), ash (3.7 wt. %) and lignin (5.9 wt. %). Sugar beet pulp has hitherto been used mainly as feed for farm animals, in the form of dry pellets or silage. The sugar beet leaves are another waste product, an estimated 120 million tons of which is produced in Europe each year. Sugar beet leaves are composed mainly of cellulose (13–18%), hemicellulose (11–17%) and pectin (14–18%), with small amount of lignin (5–6%) apart from protein and ash [3,4,5]. A more detailed analysis of the composition was made for dried beet leaves. On a dry basis, sugar beet leaves are mainly composed of structural carbohydrates (32.4%), protein (26.9%), and soluble sugars (10.0%). The structural carbohydrates are composed of polymeric sugars in nearly equal amounts of glucan (11.0%), hemicellulosic-derived sugars (xylan, galactan, and arabinan) (10.4%), and polygalacturonic acid (11.0%). In addition, the dried beet leaves consist of lignin (6.2%) and ash (19.6%) [5]. Due to their lower nutritional value, as well as the presence of saponins which cause cattle indigestion, beet leaves are used much less as fodder. Sugar beet pulp and sugar beet leaves are interesting raw materials for the chemical and biotechnological industries. Hydrolysis of hetero-polysaccharides leads to the formation monosaccharides: xylose, glucose, mannose, galactose, rhamnose and arabinose [6,7]. Hydrolysis of sugar beet pectin gives glucose, arabinose and galacturonic acid as well as smaller amounts of galactose and rhamnose [8,9,10]. The products from hydrolysis can be used as chemical raw materials or can be subjected to biological valorization.

Acidic hydrolysis is one of the most commonly-used methods for the chemical processing of biomass [11,12,13,14,15,16,17]. It is performed using a mineral acid, such as sulfuric acid [11], hydrochloric acid [12] or phosphoric acid [13], or with organic acids, mainly dicarboxylic acids [14], in concentrations of 0.5–10%. Elevated pressures and temperatures of 140–190 °C are usually required throughout the process [15,16,17]. Acid hydrolysis of waste biomass from a variety of sources including straw, bagasse, corncobs, oats and wheat bran can be used to produce furfural on an industrial scale. Due to their similar composition, sugar beet leaves and pulp, waste products of the sugar industry, could also be used. Global furfural production stands at around 300,000 tons per year [18]. The largest factories producing furfural are located in three countries: China, South Africa and the Dominican Republic. The most widely-used method of furfural production is based on the Quaker Oats process, which involves dehydration of pentosans using inorganic acid (H_2_SO_4_ less HCl). Newer continuous processes, such as the Westpro-modified Huaxia Technology or Supra Yield process, are also used. Furfural is an important renewable building block for products with high market value, such as solvents, plastics, resins and fuel additives [19,20,21,22]. Furfural and its derivatives have been used to make jet and diesel fuel range alkanes [23,24,25] and as a gasoline blendstock [26].

The current technology for producing furfural from biomass is not environmentally friendly. Attempts to reduce its environmental impact have focused on using the resulting acidic post-production leachate to produce fermentation media, after appropriate neutralization and supplementation. New technologies should not generate large amounts of effluents. Fermentation of waste, before disposal, may solve this problem [27]. Carbohydrates released during hydrolysis could be utilized by microorganisms in the process of cell proliferation, and the biomass obtained could be used as a protein supplement in animal feed [28]. Microbial biomass is defined as a form of single cell protein (SCP), and may be useful as a dietary additive. Because it contains large amounts of vitamins, lipids, proteins and all essential amino acids, SCP has high nutritional value and bioavailability [29]. The accumulation of protein in fermented waste results from the selective degradation of its components. If mineral nitrogen is provided, this is also converted to microbial biomass protein. The result is the conversion of low-quality waste biomass into a higher quality (microbial) protein source [30]. Microorganisms used in the production of SCP require special properties. They should grow rapidly, have a simple biomass production processing system, and be non-toxic and non-pathogenic. Given these requirements, the most common microbial protein additive is yeast biomass. The protein content of yeast cells does not exceed 60%, but the concentration of essential amino acids (lysine, tryptophan, threonine, and also methionine and cysteine) is satisfactory [29,31]. Bacteria, algae and fungi have also been used in the production of SCP. In the present study, both conventional and non-conventional yeast strains were cultured on the hydrolysates obtained, to identify the most suitable microorganisms for the production of biomass.

In addition to producing SCP and contributing to reduce the environmental impact of furfural production, microbiological activity can be exploited for the biosynthesis of products with industrial applications [27]. Therefore, in our study, selected lactic acid bacteria and yeasts were investigated for their ability to produce chemical products such as lactic acid and cell biomass. The main aim was to develop a concept for the processing of sugar waste (beet pulp and leaves), by simultaneous thermally-assisted acidic hydrolysis and biological processing of the sugars present in the post-production leachate. The products, single cell protein or chemical products such as lactic acid, may be used as valuable feed additives or can be catalytically valorized to propylene glycol or furfuryl and tetrahydrofurfuryl alcohol.

## 2. Results

**Figure 1:**
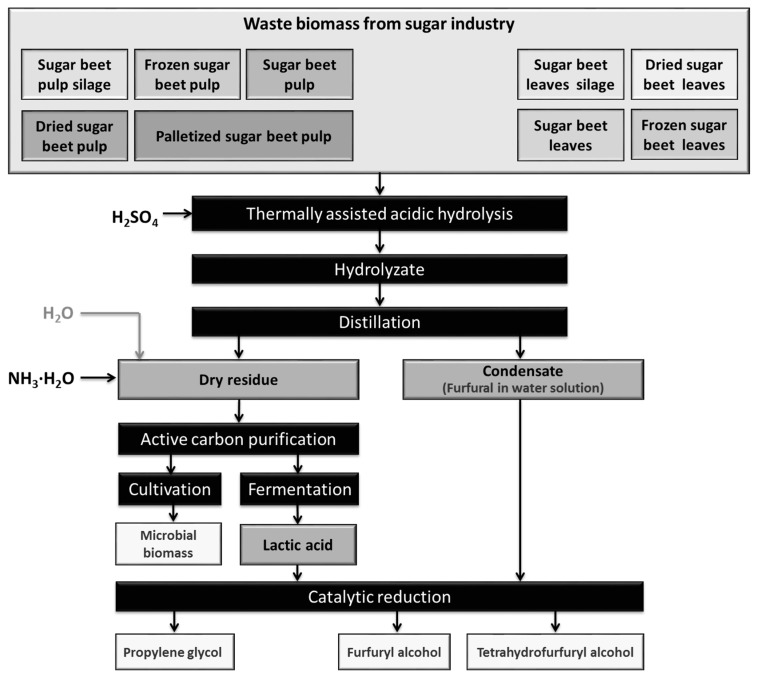
Thermally-assisted acidic hydrolysis of waste biomass with product treatments.

The processes of thermally-assisted acidic hydrolysis of waste biomass were performed according to the procedure presented in Figure 1 and described quantitatively in Section 4.2.

### 2.1. Biomass as a Source of Furfural

A method was developed for the acid hydrolysis of sugar beet pulp and sugar beet leaves, using H_2_SO_4_ acid (Figure 1). We also investigated the influence of the mode of waste biomass preservation (freezing, ensilage or drying) and of the storage time on the yield of furfural. All samples of biomass provided high furfural yields, comparable with those for fresh material (see Table 1). The yield of furfural was three times lower when leaves were used as the substratum, in comparison with sugar beet pulp. Long-term storage of ensiled waste biomass did not result in loss of furfural productivity (see Table 2). This confirms the usefulness of these methods for the commercial production of furfural.

#### Catalytic Reduction of Furfural Obtained from Biomass

Furfural (F) can be used as a raw material for the production of useful products such as furfuryl alcohol (FA), tetrahydrofurfuryl alcohol (THFA), 2-methyltetrahydrofuran (2-MTHF), 5-methyl furfural (5-MF), maleic acid (MA), tetrahydrofuran (THF) and 2-methylfuran (2-MF). In this study, we used a condensate consisting of furfural in an aqueous solution. We conducted catalytic reduction under mild temperature and H_2_ pressure conditions to obtain furfuryl alcohol and tetrahydrofurfuryl alcohol.

In the first stage, the temperature conditions, H_2_ pressure, reaction time and the amount of catalyst to be used were selected. Tests were performed using a 5%Pd/Al_2_O_3_ model catalyst and an aqueous solution of 0.1 M commercial furfural. The concentration of the solution was set at a level that could be obtained from all the condensates resulting from the distillation of the biomass hydrolysates. Based on chromatograms (obtained by Gas Chromatography-Flame Ionization Detector (GC-FID)) of the reaction mixture during the catalytic process, the concentrations of F, FA, and THFA were established. These were then used to calculate the conversion of furfural and the yields of the reaction products (FA and THFA). The activity of the catalyst was expressed in terms of furfural conversion [X%], according to the equation: X = [1 − (C/C0)] × 100%. The yields [Y%] of the main products of the reaction FA, THFA, THF, 2-MTHF were defined as: Y = [C_p_/C_0_] × 100%. In the expressions for X and Y, C_0_ is the initial furfural concentration [M]; C is the furfural concentrations at time t [M]; and C_p_ is the product concentration [M]. The results are shown in Table 3.

The optimal operating parameters for the reduction process were as follows: temperature 90 °C; hydrogen pressure 20 atm; reaction time 2 h; amount of catalyst used 0.5 g; concentration of furfural 0.1 M. The main reaction products were FA and THFA. During the reduction of furfural on 5%Pd/Al_2_O_3_ catalyst, trace amounts of 2-MTHF and THF were detected using GC-FID and GC-MS.

**Figure 2:**
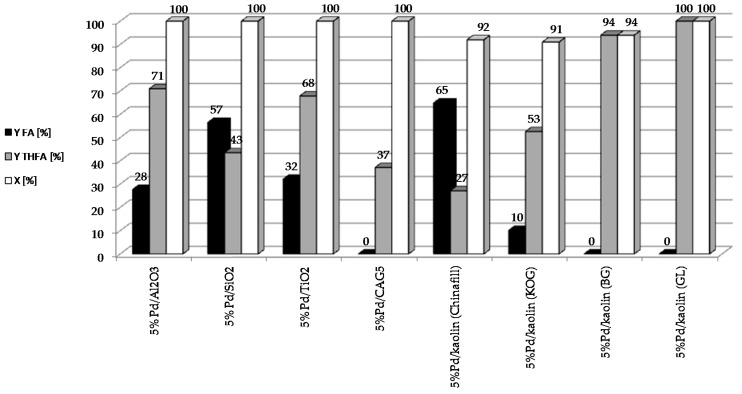
Effect of the type of support used for the reduction of furfural on the catalytic properties of heterogeneous systems containing 5 wt. % of palladium.

To determine the effect of the type of support on the catalytic properties of heterogeneous systems containing 5 wt. % of palladium, the following catalysts were prepared: 5%Pd/Kaolin_(Chinafill)_, 5%Pd/Kaolin_(KOG)_, 5%Pd/Kaolin_(GL)_, 5%Pd/Kaolin_(BG)_, 5%Pd/SiO_2_, 5%Pd/Al_2_O_3_, 5%Pd/TiO_2_, 5%Pd/C_AG5_. The reaction was carried out under constant conditions: t = 2 h, T = 90 °C, pH_2_ = 20 atm, m_cat_ = 0.5 g, C_F_ = 0.1 M. The results are shown in Figure 2.

All the palladium catalysts tested showed high activity. The rate of furfural conversion was over 90% in each of the systems studied. Interestingly, with the 5%Pd/C_AG5_ catalyst, which showed an extremely high conversion rate (over 99%), the selectivity for individual products did not add up to 100%. This may be due to the strong adsorption of the substrate or to products on the carbon support. Higher yields of THFA were obtained using systems with smaller palladium crystallites in the catalysts. In addition to FA and THFA, GC-MS analysis of the reaction mixture after catalytic measurements revealed trace amounts of 2-MTHF, THF, cyclopentanone and 4-pentenal.

**Figure 3:**
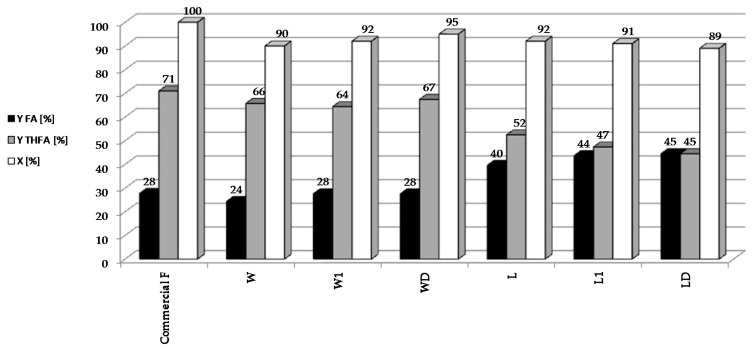
Reduction of bio-furfural (selected condensate from the distillation of biomass hydrolysates) over 5%Pd/Al_2_O_3_ catalyst under optimized conditions: t = 2 h, T = 90 °C, pH_2_ = 20 atm, m_cat_ = 0.5 g, C_F_ = 0.1 M.

In subsequent experiments, the reduction of bio-furfural (the condensate from the distillation of biomass hydrolysates) was carried out over 5%Pd/Al_2_O_3_ catalyst under optimized conditions: t = 2 h, T = 90 °C, pH_2_ = 20 atm, m_cat_ = 0.5 g, C_F_ = 0.1 M (Figure 3).

In our bio-furfural reduction studies, we have decided to use a palladium catalyst on alumina because it is well characterized by many physicochemical techniques. Furthermore, the parameters of this catalyst are similar to the commercial 5%Pd/Al_2_O_3_ system (Sigma-Aldrich, Product Number 205710, Steinheim, Germany), which we plan to use to reduce the larger amounts of bio-furfural obtained in the apparatus built in the sugar factory in Dobrzelin (Poland). It was found that the activity and yields of individual furfural hydrogenation products varied depending on the condensate used. In all of the reactions, 5%Pd/Al_2_O_3_ catalyst showed good activity (above 90%) for the reduction of furfural. However, better yields of THFA were obtained using hydrolysates of sugar beet pulp than with those from beet leaves. This may be due to the presence of trace amounts of nitrogen containing compounds in the distillates (derived from the hydrolysis of the proteins present in the leaves), which selectively chemisorb on Pd centers. According to James [32] amino acids adsorb in Pd centers. Our studies on sugar beet pulp hydrolysates showed the presence of Ala, Pro in the concentrations over 110 mmol/L; Leu, Tyr over 80 mmol/L and Asp, Glu, Gly, His, Arg, Val, Cys, Phe over 20 mmol/L [33]. Blocking the centers on the Pd surface could hinder the reduction of FA to THFA, which might explain the higher selectivity to FA observed for distillates obtained from beet leaves. These results show that FA and THFA can be obtained by catalytic reduction of furfural present in crude condensates. This is important because furfural separation and purification process is environmentally damaging, due to the use of organic solvents.

### 2.2. Biotechnological Valorization of Obtained Hydrolysates

**Figure 4:**
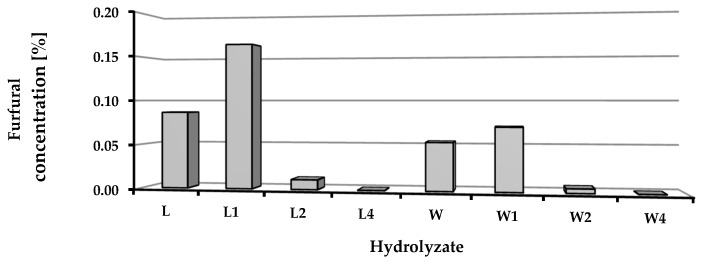
Furfural concentration in obtained hydrolysates.

The chemical composition of the hydrolysates obtained after depolymerization of the biomass was determined in terms of the content of selected monomers (glucose, fructose, mannose, arabinose, galactose, raffinose, rhamnose, xylose, and galacturonic acid) and furfural concentrations (Table 4, Figure 4). After distillation, the content of furfural in the hydrolysates was low: less than 0.16% for hydrolysates of sugar beet leaves and 0.07% for media derived from sugar beet pulp. In both cases, significantly higher levels of furfural were found in hydrolysates obtained from non-preserved and fresh ensilaged biomass (Figure 4).

The highest concentrations of mannose, arabinose and galactose (over 12 g/L) were found in sugar beet pulp hydrolysates obtained from non-preserved and fresh ensilaged biomass (W, W1). Relatively high amounts (4.2–8.8 g/L) of raffinose were also found in these hydrolysates. The content of glucose, the basic microbial carbon source, varied from 2.7 to 7.6 g/L. Media obtained from biomass after a longer period of ensilage were significantly poorer in carbohydrates. The sugar content of hydrolysates from sugar beet leaves was not particularly high. In contrast to sugar beet pulp, the highest carbohydrate concentrations, especially for glucose and raffinose, were determined after hydrolysis of beet leaf biomass which had been ensiled for three or four months.

Both the sugar beet pulp and sugar beet leaf hydrolysates were tested for their suitability as cultivation media for different industrially-used yeasts (conventional distillery yeast, bakery yeast, brewery and winery strains), as well as for non-conventional yeast belonging to the genera *Pichia, Kluyveromyces* and *Candida*. In addition, we tested their possible use as cultivation media for lactic acid bacteria, which had been found in our previous studies to be suitable for the fermentation of sugar beet pulp hydrolysates [34].

The cell proliferation process was monitored densitometrically (Table 5, Table 6 and Table 7).

The effectiveness of biomass synthesis by Saccharomyces spp. varied, depending on the type of sugar beet leaf hydrolysate used. For each type of hydrolysate, at least one yeast strain induced a change in the optical density of the medium, measured as an increase of 2 McFarland (McF). However, the best results for yeast cells biomass synthesis were observed in the case of W1 hydrolysates. These media were found to be suitable for the cultivation of almost all Saccharomyces spp. and non-Saccharomyces strains.

All the tested yeasts were capable of assimilating carbon sources from hydrolysates of both sugar beet leaves and pulp. However, the best strain was *C. utilis*, which grew very well in the tested hydrolysate media. Interestingly, *Pichia* sp. proved to be a weak producer of biomass. In terms of biomass yield, more satisfying results were achieved with unconventional yeasts. *Kluyveromyces marxianus* 179, *Kluyveromyces marxianus* 0028 and *Candida utilis* 0021 were able to grow on both types of hydrolysate, derived from sugar beet leaves and from sugar beet pulp. Relatively high yields were achieved from all sugar beet pulp hydrolysates with *Kluyveromyces marxianus* 0028 and *Candida utilis* 0021. Protein content in yeast biomass *Saccharomyces cerevisiae* and *Kluyveromyces marxianus* cells was analyzed in our previous studies [31,35]. Total protein content for different hydrolysates equaled from 231.15 ± 25.41 to 8041.95 ± 42.11 mg/L for *S. cerevisiae* and up to 3211.14 ± 132.77 mg/L for *K. marxianus*. Thus, these microorganisms can be a valuable source of proteins.

The differences in terms of the utility of different types of tested media were not as great for lactic acid bacteria as in the case of the yeast strains. However, the best biomass yield was observed for *Lb. brevis* 488 cultured on W and W1 sugar beet pulp hydrolysates. With all the tested lactic acid bacteria, the greatest increase in optical density occurred during incubation on W1 media.

**Figure 5:**
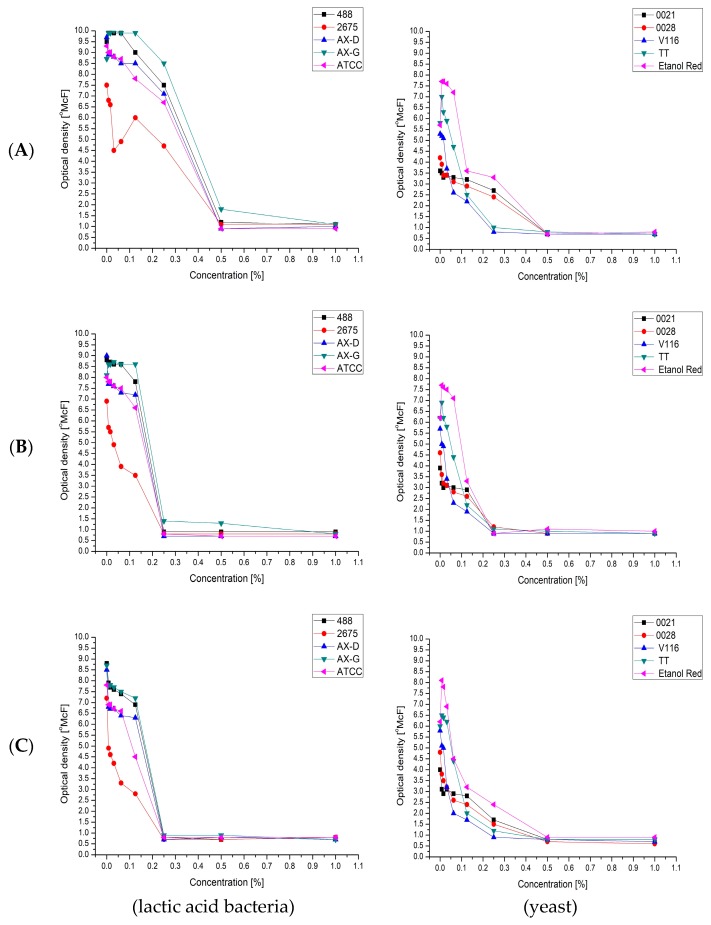
Minimal inhibitory concentration of: furfural (**A**); vanillin (**B**); and levulinic acid (**C**); for selected: lactic acid bacteria and yeast strains.

The second stage of the biomass proliferation process was conducted after the addition of sterile water, which diluted all the compounds in the media (including the carbohydrates). Despite this, in many cases further growth was observed. With some strains and media, proliferation began after the addition of water. This suggested the presence of growth inhibitors. The effect of known growth inhibitors derived from lignocellulosic biomass—furfural (Figure 5A), vanillin (Figure 5B) and levulinic acid (Figure 5C), in concentrations ranging from 0.0078% to 1%—was therefore investigated, using the densitometric method.

The effect was measured as the difference between the optical density (OD) measured after 24 h (OD24h) and that just after inoculation. The results showed that lactic acid bacteria were the most sensitive to vanillin and levulinic acid, with Minimal Inhibitory Concentration (MIC) values of 0.25%. For furfural, the MIC value was 0.5%. The environmental strain *Lactobacillus plantarum* AX-G was the least sensitive to the tested chemical compounds, while the strain that showed the least resistance was *Lactobacillus plantarum* 2675.

**Figure 6:**
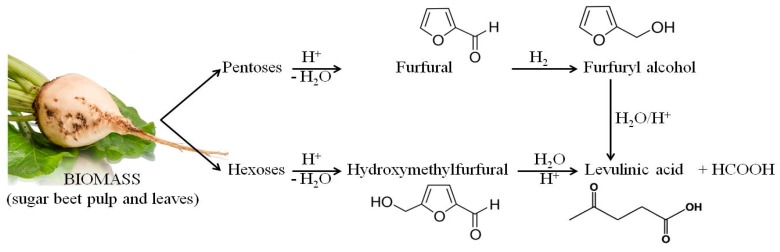
Products of acid hydrolysis of waste biomass from the sugar industry.

As in the case of lactic acid bacteria, the chemical compound that exhibited the strongest inhibitory activity against the tested strains of yeasts was vanillin. The use of 0.25% of this compound clearly inhibited the growth of all of the tested yeast strains. Furfural and levulinic acid concentrations of 0.25% inhibited the growth of *Saccharomyces cerevisiae* TT and *Saccharomyces cerevisiae* V116. The minimum concentration of furfural that inhibited the growth of *Candida utilis* 0021, *Kluyveromyces marxianus* 0028 and *Saccharomyces cerevisiae* Ethanol Red was 0.5%. It is also important to note the similarity between the MIC results for furfural and levulinic acid. This may be because levulinic acid and furfural are derived from sugars (pentoses or hexoses) which are produced by acidic hydrolysis of biomass (sugar beet pulp and leaves). Moreover, levulinic acid can be obtained from furfural (Figure 6).

#### Catalytic Reduction of Lactic Acid

**Figure 7:**
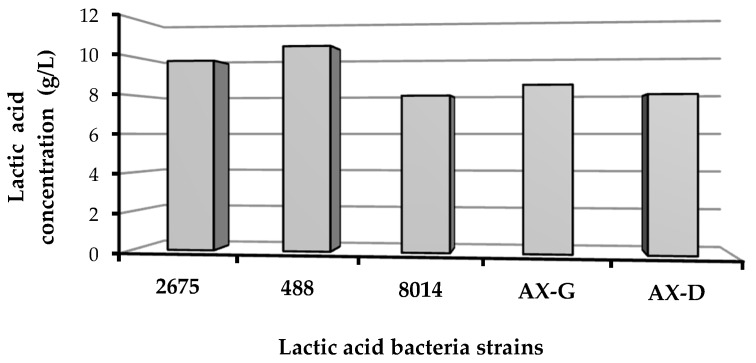
Lactic acid productivity of bacterial strains cultured on W1 hydrolysate.

Based on the carbohydrate profiles of the biomass hydrolysates and the absence of a strong inhibitory effect, W1 was selected for lactic acid biosynthesis. As shown in Figure 7, all tested strains (*Lactobacillus plantarum* 2675, *Lactobacillus brevis* 488, *Lactobacillus plantarum* 8014, *Lactobacillus plantarum* AX-G and *Lactobacillus plantarum* AX-D) were able to acidify W1 hydrolysate. The lactic acid content varied from 7.93 to 10.49 g per liter of post-fermentation medium.

**Figure 8:**
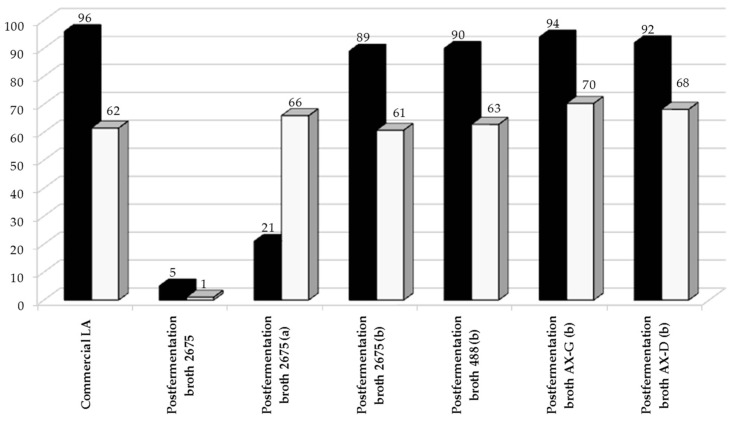
Catalytic conversion of lactic acid in post-fermentation media purified to various degrees (■—X (%)) and selectivity to propylene glycol (□—S_PG_ (%)). Lactic acid was obtained through biological synthesis from sugar beet hydrolysates. The reaction was conducted over 5%Ru/C catalyst under optimized conditions: t = 4 h, T = 130 ° C, pH_2_ = 35 atm, m_cat_ = 0.5 g, C_LA_ = 0.1 M. a, 50 mL of fermentation broth after purification on 2 g of C_act_; b, 50 mL of fermentation broth after purification on a mixture of 2 g of C_act_ and 2 g SiO_2_.

Lactic acid (LA) is one of the primary platform chemicals, and can be used to synthesize a wide variety of useful products. The catalytic transformation of LA into propylene glycol (PG) over metal-based heterogeneous catalysts is of great interest to industry, since currently this product is produced from petrochemicals. Biologically-obtained lactic acid may be used as a substrate in propylene glycol production, but only after purification on a mixture of active carbon and silica [33,36]. The effect of impurities on PG formation over 5%Ru/C catalyst is shown in Figure 7. An untreated sample of the *Lactobacillus plantarum* 2675 post-fermentation media was catalytically reduced, along with samples that had undergone purification on active carbon (ERCARBON GE, 3 g/50 mL) or on a mixture of active carbon and silica (POCH Gliwice SA, 5 g/50 mL) (Figure 8). The results reveal that catalytic reduction of the broths was sufficient to enable the effective conversion of LA into PG.

## 3. Discussion

### 3.1. Dehydration of Pentoses into Furfural and Catalytic Reduction into Furfural Alcohol and Tetrahydrofurfural

**Figure 9:**
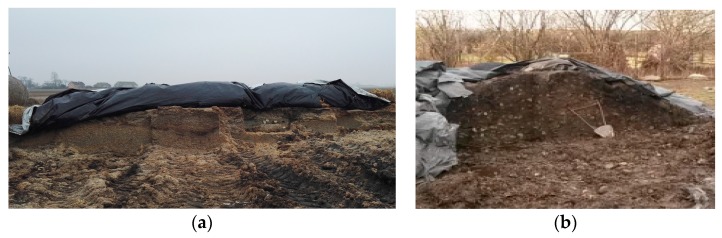
Traditional method of silage in prisms of: (**a**) beet pulp; and (**b**) beet leaves.

Biomass is considered by some to be the only sustainable source of energy and organic carbon for industry, with the potential to displace petroleum in the production of chemicals and liquid transportation fuels [37]. Economical and energy-efficient processes are required for the sustainable production of so-called platform chemical compounds—chemicals that can be easily converted into other valuable chemicals. Furfural, one of the main products of the acid hydrolysis of biomass, is often cited as a chemical platform compound for the production of chemicals and fuels [38,39,40,41]. Furfural contains both a carbonyl group, which can undergo reduction, oxidation, acylation, acetylation, aldolization, Knoevenagel condensation, decarboxylation and Grignard reactions, and a furan ring, which can undergo hydrogenation, oxidation, alkylation, halogenation, ring opening and nitration [38,39,40,41]. However, if furfural is to be used as raw material, it must first be separated from the water phase and purified. These processes have a great impact on both the price of furfural and (because they require organic solvents) also on the environment. The development of new aqueous phase processes, such as that presented in this article, is therefore very desirable from both an economic and environmental standpoint. Liquid-phase hydrogenation of furfural over catalysts based on Cu [42,43], Ni [44], on noble metals such as Ru and Rh, or on Pt [45], can yield valuable products including FA, THFA, 2-MF and 2-MTHF. The influence of the type of support on the catalytic properties of metallic systems for the reduction of furfural requires more comprehensive studies. The most common supports that have been considered for palladium catalysts used in this reaction include simple oxide supports such as SiO_2_ [40], Al_2_O_3_ [43] and MgO [42], or activated carbons [42]. In our investigation, the process of furfural hydrogenation performed over palladium catalyst supported on various mineral carriers (Al_2_O_3_, SiO_2_, TiO_2_, C_AG5_, Kaolins). Natural kaolins provide particularly interesting supports. The chemical composition of these clay minerals is Al_2_Si_2_O_5_(OH)_4_, in which one tetrahedral sheet of silica (SiO_4_) is linked through oxygen atoms to one octahedral sheet of alumina octahedra (AlO_6_). We used four kaolin carriers extracted from various mines: Grudzień Las (GL); Biała Góra (BG); the Surmin Mine (KOG); and Chinafill. The carriers differed significantly in terms of phase composition (Figure 9) as well as surfaces area (Table 8).

Therefore, it was assumed that the palladium deposited on these systems would be characterized by various degrees of dispersion, and that these systems would exhibit variable catalytic properties. The highest yield of FA was observed in the case of 5%Pd/SiO_2_, in which the Pd crystallites are larger (diameter approximately 40 nm). In the case of palladium catalysts with smaller Pd crystallites (7–10 nm), THFA was the main product of the reaction. Understanding the nature of this phenomenon is important for the creation of new and stable Pd-based catalysts for the reduction of furfural.

### 3.2. Utilization of Carbohydrates by Selected Microorganisms

Usually, classical yeasts from the genus *Saccharomyces spp.* are unsuitable for the assimilation of carbon sources from hydrolysates, since they are unable to ferment pentoses and exhibit low tolerance to alcohols, acids and solvents. Moreover, they are characterized by high sensitivity to both pH changes and cytotoxic compounds, including furfural, 5-(hydroxymethyl)-2-furfural and other organic compounds. The limitations of *S. cerevisiae* make the development of new industrial fermentation processes very difficult. An additional problem for the simultaneous consumption of pentoses and hexoses is that pentose uptake is inhibited by d-glucose. Progress in improving the utilization of pentose sugars has been slow, as there are few yeasts known to be capable of metabolizing pentose. Therefore, our interest was directed towards using both industrial strains and yeasts belonging to genera other than *Saccharomyces*, commonly referred to as “non-conventional” yeasts.

The use of non-conventional yeasts may overcome many problems related to the narrow spectrum of carbon sources assimilated by conventional *S. cerevisiae* [46]. Some non-conventional yeast show many uncommon, metabolic features of potential interest to biotechnologists. In *S. cerevisiae*, monosaccharides such as glucose, fructose or mannose are transported in a facilitated diffusion process. However, the process of transportation may be different in other yeasts. For example, in *Kluyveromyces* spp., glucose transport appears to proceed by facilitated diffusion. When *Candida utilis*, the popular “fodder yeast”, is grown at low glucose concentrations, the glucose appears to be transported by proton transport [47]. Non-conventional yeasts comprise the vast majority of genera and species so far described. Several yeast species have evolved from *S. cerevisiae* but possess several unique genes and growth characteristics that make them capable of withstanding different stress conditions [48]. These exceptional strains are able to utilize various sources of carbon, such as starch, cellulose, raffinose, arabinose, xylose and sugar alcohols. In our study, hydrolysates rich in mannose, arabinose, galactose and raffinose were found to be suitable for use as cultivation media for *Kluyveromyces marxianus* and *Candida utilis*. These yeasts showed good growth on both beet leaf and beet pulp hydrolysates. The yeast *Candida* sp. has been used for many years as a microbe of choice in the production of SCP from media containing carbohydrates. *C. utilis* is used with a wide variety of substrates, such as sucrose, ethanol and sulfite-spent liquor. Because of its ability to assimilate pentoses, it can also grow on wood hydrolysates [49]. *K. marxianus* can grow on lactose, inulin (a fructose polymer found in some plants) and other simple sugars such as glucose, fructose and sucrose. It is sometimes therefore also grown on molasses [50].

Dilute-acid hydrolysis is fast and easy to perform, but is hampered by non-selectivity and the formation of by-products. The composition and concentration of inhibitory compounds varies depending on the type of lignocellulosic raw material and how it is pre-treated [51]. Generally, after pre-treatment of the hemicellulose fraction, hexoses (d-glucose, d-galactose, d-mannose and d-rhamnose) and pentoses (d-xylose and l-arabinose) are obtained [52,53]. However, under high temperature and pressure conditions, hexoses and pentoses may be degraded to 5-(hydroxymethyl)-2-furfural and furfural. In general, while furfural and 5-hydroxymethylfurfural can originate from the dehydration of pentoses and hexoses, vanillin originates from degradation of lignin polymers. Thus, in the case of relatively sugar-rich hydrolysates obtained from sugar beet pulp, the potential inhibitors of microbial growth are mainly furfural, HMF and levulinic acid, while vanillin tends to be present mainly in beet leaves pulp. However, due to the fact that in both cases it is possible to obtain a mixture of compounds inhibiting microbial growth it is justified to examine the influence of these inhibitors on the tested microorganisms [54]. Furthermore, furans and phenol derivatives such as vanillin can be formed in low concentration, what with the presence of other compounds makes them difficult to analyze. Although their presence is low in the medium, they may be highly toxic for the growth and fermentation of microorganisms [55]. Therefore, it seems to be very important to determine the susceptibility of the microorganisms to these compounds.

Phenolic compounds are generated from the partial breakdown of lignin and have also been reported as forming during the degradation of carbohydrates [52]. The harmful effects of these compounds, even at low concentrations, have been confirmed. The RNA and DNA, proteins and membranes of yeast cells are particularly sensitive [56,57]. Phenolic compounds partition into biological membranes, causing loss of integrity and affecting their ability to serve as selective barriers and enzyme matrices. Vanillin constitutes a large fraction of the phenolic monomers in biomass hydrolysates. Furfural has been shown to reduce the specific growth rate and the cell-mass yield of yeasts [52]. In our research, the level of furfural in the hydrolysates following distillation did not exceed 0.16%. Despite this fact, an inhibitory effect was observed in some cultures. This can be explained by the synergistic action of several inhibitors. Combinations of different by-products can have synergistic inhibitory effects on the growth of yeast strains [58]. Each of the tested strains was found to be sensitive to all three investigated compounds. Furfural has been shown to have a negative impact on yeast growth (observed as a decrease in the specific growth rate). Its action in the presence of mixed microbial inhibitors can be greater than that of any of the individual compounds [52]. Most studies on the toxicity of by-products have focused on ethanol-producing yeasts. The toxicity of by-products on lactic acid producing strains has received less attention. In research conducted by van der Pol et al., the inhibition of lactic acid bacteria growth showed large inter-species variation. This variation was confirmed in our study [58].

Removing toxic compounds from the fermentation medium is usually very expensive. It is therefore more practical to use furan-tolerant yeast strains. According to the literature, some non-conventional yeast species, especially *Pichia* spp. and *Candida* spp., show good tolerance to furfural and its derivatives. For example, the resistance of *P. kudriavzevii* to hydroxymethyl can be up to 7 g/L [59]. *Candida utilis* was confirmed in our study to exhibit good growth in sugar beet hydrolysates.

### 3.3. Waste Biomass from the Sugar Industry as a Potential Source of Platform Chemicals (Furfural, Lactic Acid) and Biotechnological Media

Waste lignocellulose residues, including waste biomass from the sugar beet industry, is usually hydrolyzed for the purposes of the production of second generation fuel, mainly bioethanol [60,61]. Here, we present an alternative solution, providing as end products furfural, furfuryl alcohol, tetrafurfuryl alcohol, lactic acid and propylene glycol.

Hydrolysis involves the treatment of lignocellulose at high temperatures, under acidic conditions. It leads to the formation and liberation of a range of compounds. When hemicellulose is degraded, xylose, mannose, acetic acid, galactose and glucose are liberated. Cellulose is hydrolyzed to glucose. At high temperatures and under pressure, xylose is further degraded to furfural [52]. The conditions of hydrolysis—residence time, temperature and acid concentration—have a strong effect on the spectrum of products. As the values for these parameters increase, the yield of fermentable sugars also increases, to a maximum point above which it starts to decrease. The decrease in the yield of monosaccharides coincides with the maximum concentrations of furfural [62]. This requires a proper balance to be found between the production of monosaccharides and of furfural.

The bio-based media were next used for the production of propylene glycol from the lactic acid biosynthesized in the hydrolysate. The process of bioconversion of sugar beet pulp carbohydrates into lactic acid has been described in several previous studies [33,34,36]. Lactic acid is one of the primary platform chemicals, and can be used to synthesize a wide variety of useful products. Its reduced derivatives, such as propylene glycol, can be used as green chemicals in industrial applications [34]. The reduction of lactic acid to propylene glycol was conducted over commercial ruthenium catalysts under mild temperature conditions (130 °C) and H_2_ pressure (35 atm). In our earlier studies, these conditions had been found to be optimal [33,36]. As can be seen in Figure 7, the lactic acid obtained from the sugar beet pulp hydrolysates can be converted with good yields into propylene glycol. However, this requires initial purification of the media, since the sulfide-containing amino acids present in post-fermentation broths irreversibly poison the ruthenium catalysts, whereas non-sulfurous amino acids only partially and reversibly poison the catalysts [33]. In the conventional biological method of pure lactic acid production, the separation and purification stages account for up to 50% of production costs. In our study, the post-fermentation broth was purified using active carbon. A diluted water solution of the lactic acid was then reduced over a Ru-based supported catalyst. The yield of propylene glycol obtained in this way was satisfactory, and the water solutions of propylene glycol obtained could be used in concentrated form as a component in anti-freeze. The process of propylene glycol production described here could be of interest to industry, but its disadvantage is that it is material intensive (requiring 1.25 kg LA/1.00 kg PG). It is therefore necessary to the reduce costs and increase efficiency. The use of less pure LA as a feedstock is one possibility.

Our research clearly shows that the hydrolysates obtained from waste products of the sugar industry provide a good carbon source for the selected yeasts, especially the non-conventional yeast strains. Production systems exploiting some non-*Saccharomyces* yeasts have a distinct advantage, in that they are non-pathogenic and have received the “generally recognized as safe” (GRAS) designation from the FDA [35,63]. Yeast species other than *Saccharomyces* spp. use complex substrates, and exhibit features such as thermotolerance and tolerance to the presence of chemical inhibitors, making them particularly useful in industrial processes. For example, its broad enzymatic activity and fermentation ability in the presence of high concentrations of saccharides makes the yeast *K. marxianus* a good material for various biotechnological processes [64,65]. *K. marxianus* cultures have also been grown on sugar beet leaf hydrolysates, and *Candida tropicalis* on media derived from both sugar beet pulp and sugar beet leaf hydrolysates, with positive results [66,67]. The advantages of the solution presented here include not only the value of the end products but also the fact it enables virtually “zero-waste” production. Currently, the sugar industry produces large amounts of waste biomass, only part of which can be only used without processing, as components in animal feed [68,69].

## 4. Materials and Methods

### 4.1. Biological Material

#### 4.1.1. Lignocellulosic Plant Material

Sugar beet pulp and beet leaves provided the lignocellulosic plant material for the hydrolysates (Table 9). These wastes from sugar production processes were obtained from a sugar factory located in Dobrzelin. The raw material was first examined and then subjected to storage processes: drying, ensilage or freezing. Samples of fresh sugar beet pulp and beet leaves obtained from the Dobrzelin Sugar Factory were dried in a laboratory oven at 110 °C to a constant weight and stored in a desiccator above the drying layer until testing. For comparison, hydrolysis of sugar beet pulp was performed using pelletized samples which had been dried in the sugar factory according to industrial procedures. Other samples of fresh sugar beet pulp and beet leaves obtained from the Dobrzelin sugar factory were frozen in industrial food freezers at approximately −7 °C. They were stored at the same temperature before being thawed at room temperature immediately prior to hydrolysis.

Further samples of beet pulp and leaves were ensiled according to the traditional method from November 2016 to May 2017, while the first sample was collected one month after. Figure 9 shows beet pulp and beet leaf prisms prepared on a farm. Each prism of beet pulp/beet leaves (8.0 × 3.0 × 1.5 m) was insulated with black rubber foil and sealed using tires to cut off air and maintain appropriate temperature conditions. Samples of the biomass were collected at fixed intervals and examined immediately upon delivery to the Institute of General and Ecological Chemistry.

##### Determination of Dry Matter

To determine the amount of dry matter in the biological samples, their relative humidity was measured by heating each sample to 100–105 °C and recording the mass lost due to evaporation. Measurements were made on a Radwag MA moisture analyzer, with an IR radiator used as the heat source. The samples of plant material were weighed before heating and again once they had reached a constant weight. Based on the differences in the weights of the samples, the amount of dry matter was calculated.

#### 4.1.2. Yeast Strains

Ten collection strains were used: *Pichia angusta* NCYC495, *Kluyveromyces marxianus* NCYC179 *Saccharomyces pastorianus* NCYC203, *Saccharomyces cerevisiae* NCYC1183 and *Saccharomyces pastorianus* NCYC1116 from the National Collection of Yeast Cultures (UK); *Kluyveromyces marxianus* LOCK0028, *Candida utilis* LOCK0021, *Saccharomyces cerevisiae* TT LOCK0271A, *Saccharomyces cerevisiae* JA64 LOCK0134 and *Saccharomyces cerevisiae* Tokay LOCK0204 from the Lodz Culture Collection (Poland) and two commercial strains, *Saccharomyces cerevisiae* Ethanol Red (Fermentis Division S.I. Lesaffre, France Fermentis Division S.I., Lesaffre, Marcq-en-Baroeul, France) and *Saccharomyces cerevisiae* Lalvin V1116 (Lallemand Inc., Montreal, QC, Canada).

#### 4.1.3. Lactic Acid Bacteria Strains

The following lactic acid bacteria strains were used: *Lactobacillus plantarum* 2675 (Polish Collection of Microorganisms), *Lactobacillus brevis* 488 (Polish Collection of Microorganisms), *Lactobacillu splantarum* 8014 (American Type Culture Collection), *Lactobacillus plantarum* AX-G (environmental isolate with accession number KT 751285 in the NCBI GenBank), *Lactobacillus plantarum* AX-D (environmental isolate with accession number KT 751284 in the NCBI GenBank).

### 4.2. Acid Hydrolysis of Biomass

Each analyzed biomass was weighed on an analytical balance, and a sample corresponding to 25 g of dry matter was placed in a 1 L round bottom flask, to which was added 40 mL of sulfuric acid (VI) (95% H_2_SO_4_, p.a., Chempur) and 210 mL of deionized water. The mixture in the flask was heated to boiling point and distilled. The temperature of the heating cap was set to 140 °C. The distillation process was halted when the temperature of the steam at the top of the flask exceeded 100 °C. The distillates were collected and subjected to GC-FID and GC-MS. After neutralization (pH = 7) with saturated sodium carbonate solution (Na_2_CO_3_, p.a., POCh Gliwice SA), samples of the distillates were subjected to catalytic testing.

To determine the content of sugars in the hydrolysates, 100 mL of deionized water was added to the residue in the distillation flasks. The resulting samples were filtered using analytical medium-thickness filters to separate the solids and then alkalized with ammonia (25% NH_4_OH, p.a., Chempur) to pH 5.5. The hydrolysates were then purified on activated carbon (2 g, Ercarbon GE, ERBSLÖH Geisenheim AG, Geisenheim, Germany) to remove organic impurities and re-filtered to separate the adsorbent.

### 4.3. Catalytic Research

#### 4.3.1. Furfural Reduction

The reaction conditions were optimized for monometallic Pd catalysts. The mass-transfer limitations were determined experimentally using diagnostic criteria: varying the stirring speed (50–750 rpm), catalyst concentration (0.1–2 g·L^−1^) and particle size (0.5–0.075 mm). All the liquid phase reactions of the furfural solution (0.1 M·L^−1^,Sigma Aldrich, 99%) were performed in a high pressure steel reactor (Paar 4590), at a constant temperature of 90 °C, under 20 atm H_2_ pressure (Air Products, Premium Plus, 99.999%). Before the measurements were taken, the catalyst and liquid substrate were placed in the reactor and the whole system was purged with argon (Ar, Linde 5.0) at a rate of 20 mL·min^−1^ at 20 °C for 15 min, and then with hydrogen at 20 °C for 15 min. The reactor valves were then closed and the hydrogen pressure raised to 20 atm. Next, the temperature of reaction mixture was gradually raised to 90 °C. The linear temperature increase rate was 20 °C·min^−1^. The reaction was continued for two hours.

The results reveal the influence of mass transfer on the liquid/solid interface and of intra-particle diffusion in limiting the rate of furfural hydrogenation. However, these effects were not observed when the reaction mixture was stirred at least 400 rpm, when the catalyst concentration was above 0.2 g·L^−1^ and when the particle size of the catalysts was less than 0.15 mm. Each experiment was conducted with an equal amount of catalyst (m_cat_ = 0.5 g). All reactions over monometallic catalysts were therefore performed under the conditions described below to eliminate diffusion restrictions.

**Figure 10:**
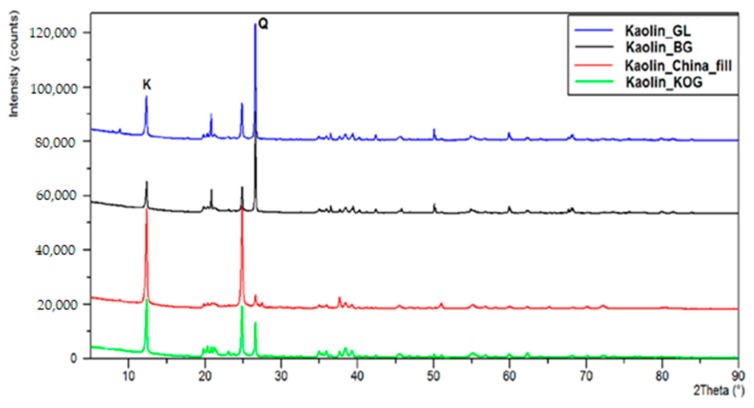
Diffractograms of kaolin carriers sourced from various mines.

All the materials (Al_2_O_3_, SiO_2_, TiO_2_, C_AG5_, Kaolins) used as supports in the palladium catalysts remained inactive during the process of furfural hydrogenation. The specific surface area and textural properties of each of the studied supports are summarized in Table 8. XRD diffractograms were used to determine the phase composition of the kaolin carriers sourced from various mines (Figure 10). On the diffraction patterns, the letter K denotes the main diffraction lines corresponding to the crystalline kaolinite phase Al_4_[Si_4_O_10_](OH)_8_, while the letter Q is the main diffractive peak corresponding to quartz SiO_2_. In kaolin extracted from the Grudzeń Las and Biała Góra mines, the main crystalline phase was quartz. The kaolins supplied by the Surmin mine (KOG and Chinafill) contained large quantities of kaolinite. Thus, the kaolins differed significantly in terms of their phase composition and surfaces areas. It was assumed that palladium deposited on these systems would be dispersed differently, and that the systems would therefore exhibit various catalytic properties.

Catalysts containing 5 wt. % of palladium were prepared by aqueous impregnation of the supports: Kaolin (Chinafill, Hirschau, Germany), Kaolin (KOG), Kaolin (GL), Kaolin (BG), SiO_2_ (Sigma Aldrich), Al_2_O_3_ (Fluka), TiO_2_ (POCh Gliwice S.A., Gliwice, Poland) and C_AG5_ (Hajnówka, Poland). A solution of PdCl_2_ (POCH, anhydrous, pure p.a.) acidified to a pH of around 5 with HCl_aq_ (CHEMPUR, Karlsruhe, Germany, 35–38%, pure p.a.), was used. The water was evaporated at an elevated temperature (T = 60 °C) under a vacuum. The monometallic catalysts were dried in air at 110 °C for 6 h, calcined at 500 °C for 4 h in an oxygen atmosphere (O_2_, Air Products, 99.5%) at a rate of 20 mL·min^−1^, cooled in argon to room temperature (Ar, Linde 5.0) at a rate of 20 mL·min^−1^, and then reduced in a hydrogen atmosphere (H_2_, Air Products, Premium Plus, 99.999%) at a rate 20 mL·min^−1^ for 2 h at 300 °C before catalytic measurements were taken. The linear temperature increase rate between the thermal processing steps was 20 °C·min^−1^.

#### 4.3.2. Propylene Glycol Synthesis

Hydrogenation of lactic acid was performed in a 50 mL autoclave (Parr Company, Moline, IL, USA) at 130 °C under 35 atm of H_2_ pressure. The reactions were conducted with equal amounts of catalyst (m_cat_ = 0.5 g). The mixture was stirred at 500 rpm. The autoclave was flushed with Ar, then flushed again with H_2_ and pressurized with H_2_ to 35 atm. The temperature was gradually raised to 130 °C at a heating rate of 20 °C·min^−1^. The reaction was sustained for 4 h. After the reaction, the autoclave was cooled to room temperature. The reaction mixture was filtered then analyzed using HPLC and GC-FID. Selectivity for propylene glycol was determined using the Equation:

$$S_{PG}=\left[\frac{C_{PG}}{\left(C_{0}-C\right)}\right]\times 100\%$$

where $C_{PG}$ is the concentration of propylene glycol [M]; $C_{0}$ is the initial concentration of lactate ions [M]; and C is the concentration of lactate ions at time t [M].

### 4.4. Preparation of Hydrolysate and Proliferation of Microbial Biomass

To prepare the hydrolysate for cultivation of yeast and bacteria, 100 mL of deionized water was added to the residue in the distillation flask. The resulting samples were filtered using analytical medium-thickness filters to separate the solids and then alkalized with ammonia (25% NH_4_OH, p.a., Chempur) to pH 5.5. The hydrolysates were then purified on activated carbon (2 g, Ercarbon GE, ERBSLÖH Geisenheim AG) to remove the organic impurities and re-filtered to separate the adsorbent. Biological tests were then conducted in glass tubes, to which 5 mL of each tested medium was added. All tubes with hydrolysates were sterilized at 121 °C for 15 min.

#### 4.4.1. Yeast Cultivation

The sterile media were inoculated with 0.3 mL of prepared yeast cells suspensions cultured in MEB medium (Merck Millipore, Bedford, MA, USA) and incubated at 30 °C for 72 h. To adapt the cells to the hydrolysate-medium, at least 3 sequential passages were conducted. The experiment was performed in two stages. In the first stage, the samples were incubated for 72 h. In the second stage, 5 mL of sterile water was added to each of the cultures (the hydrolysate was diluted 2-fold) and incubation was continued for another 48 h. Yeast cell growth was monitored in terms of optical density every 24 h using a DEN-1 densitometer (Merck-Millipore). At least 3 sequential passages were conducted. The results were reported in McFarland units.

#### 4.4.2. Cultivation of Lactic Acid Bacteria

Sterile samples of media were inoculated with 0.2 mL of lactic acid bacteria suspensions cultured in MRS medium (Merck Millipore), and then incubated at 37 °C for 72 h. To adapt the cells to the hydrolysate-medium, at least 3 sequential passages were conducted. The experiment was carried out in two stages. In the first stage, the samples were incubated for 72 h. In the second stage, 5 mL of sterile water was added to each culture (the hydrolysate was diluted 2-fold) and incubation was continued for another 48 h. Yeast cell growth was monitored in terms of optical density every 24 h using a DEN-1 densitometer (Merck-Millipore). At least 3 sequential passages were conducted. The results were reported in McFarland units.

### 4.5. Minimal Inhibitory Concentrations (MIC) of Furfural, Vanillin and Levulinic Acid

The minimal inhibitory concentrations (MIC) of furfural, vanillin and levulinic acid were determined using standard methods for the evaluation of disinfectant activity, according to the relevant PN EN standards. The MICs for bacterial growth were measured in accordance with PN-EN 1040: 2006 “Chemical disinfectants and antiseptics—Quantitative suspension test for the evaluation of basic bactericidal activity of chemical disinfectants and antiseptics. Test method and requirements”. The MICs for yeast growth were measured in accordance with PN-EN 1275:2006 “Chemical disinfectants and antiseptics—Quantitative suspension test for the evaluation of basic fungicidal or basic yeasticidal activity of chemical disinfectants and antiseptics. Test method and requirements”.

To prepare inoculum suspensions of the bacterial strains, 24 h cultures were centrifuged at 6500 rpm for 5 min at 22 °C (5804 R Eppendorf, Hamburg, Germany). The supernatant was discarded and the biomass was re-suspended in tryptone water (sodium chloride 8.5 g/L; tryptone 1 g/L). The suspension was then standardized, the number of cells approximately 3.0 × 10^8^/mL. ADEN-1 densitometer (Merck-Millipore) was used to set the optical density of the inoculum to 0.5 McF (the value corresponding to 3.0 × 10^8^ cells/mL). Inoculum suspensions of the yeast strains were prepared using a Thoma cell counting chamber, so that the number of yeast cells was approximately 3.0 × 10^7^/mL.

The MICs of chemicals inhibiting microbial growth were determined as follows. First, 1 mL of one of the tested chemicals (furfural, vanillin or levulinic acid) was added to a tube containing 1 mL of the appropriate medium, with either the test microorganism MRS (Merck Millipore) for lactic acid bacteria or MEB (Merck Millipore) for yeasts. A series of 2-fold dilutions were then performed. To each tube with diluted chemical agent, 1 mL of previously prepared inoculum was added. The concentration ranges of the tested chemical compounds were found to be between 0.0078% and 1%. The control was a medium without the addition of a growth inhibitor. The samples were incubated at appropriate temperatures: 37 °C for lactic acid bacteria or 25 °C for yeasts. After 24 h of incubation, the turbidity of each sample was measured using a DEN-1 densitometer (Merck Millipore). The results were reported in McFarland units.

### 4.6. Lactic Acid Fermentation of Selected Hydrolysates

Fermentation was conducted in 100 mL Erlenmeyer flasks filled with 50 mL of sterile medium supplemented with mineral and nitrogen compounds (yeast extract 0.16%, beef extract 0.33%, peptone K 0.42%, triammonium citrate 0.08%, dipotassium hydrogen phosphate 0.08%, sodium acetate 0.2%, magnesium sulfate × 7 H_2_O 0.08%, manganese sulfate × 4 H_2_O 0.002%—doses per sugar beet pulp dry mass). The hydrolysates inoculated with lactic acid bacteria were incubated at 37 °C for 48 h.

### 4.7. Analytical Methods for Liquid Media

#### 4.7.1. Chromatographic Analysis of Distillates and Reaction Mixtures

The furfural distillates and reaction mixtures after catalytic reduction of furfural were subjected to GC-MS (PerkinElmer GC–MS; Clarus 580 with MS Clarus SQ 8 S; Elite-5MS capillary column: 30 m length, 0.25 mm i.d. and 0.5 m film thickness) and GC-FID (Hewlett Packard 5890 Series II gas chromatograph equipped with a flame-ionizing detector and capillary column (Restek RTX—5: 30 m × 0.53 mm × 1.50 μm, Helium (Linde; 99.999%)). The operating conditions for the GC-FID analysis were as follows: flow rate of the carrier gas 30 mL/min; dispenser temperature 150 °C; column temperature 60–150 °C; initial temperature 60 °C; start time 2 min; temperature rise 2 °C/min; detector temperature 250 °C; injection volume 1 μL. The main validation parameters are summarized in Table 10.

An HPLC system (LaChrome, Merck-Hitachi) coupled to a variable wavelength UV (210 nm) detector was used to determine the concentration of lactic acid. The reactant was separated on an Agilent ZORBAX SB- C18 column, using a mobile phase acetonitrile/phosphate buffer (12:88 *v/v*, pH = 4.5, C = 0.01M). The concentration of propylene glycol was determined using GC-FID (Hewlet Packard 5890A; Restek RTX 5 column). The operating conditions for the GC-FID analysis were as follows: injection port temperature 70 °C; FID detector temperature 250 °C; column oven temperature 150 °C; temperature increase 15 °C·min^−1^, carrier gas He (Linde, 99.999%, flow rate 30 mL·min^−1^); injection volume 1 µL.

#### 4.7.2. Concentration of Monosaccharides in the Hydrolysates

The monosaccharide profiles of the obtained hydrolysates were analyzed using UV-spectrophotometry and Megazyme Kits (Megazyme, Inc.; County Wicklow, Ireland). The K-MANGL kit was used for glucose, mannose and fructose; the K-ARGA kit for arabinose; the K-URONIC kit for galacturonic acid; the K-XYLOSE kit for xylose; the K-RAFGA kit for raffinose; the K-RHAMNOSE kit for rhamnose.

### 4.8. Physico-Chemical Methods for Analyzing Catalyst Structure

#### 4.8.1. Low-temperature Adsorption/Desorption of N_2_ measurements

The specific surface area (SSA) of the samples was determined using Micromeritics ASAP 2020 equipment. The analysis was based on the BET model of N_2_ low temperature adsorption with the assumption that nitrogen molecules covered 0.162 nm^2^ of the adsorbent surface. The size and volume of pores with radiuses of between 0.85 nm and 150.00 nm were determined using BJH desorption cumulative volume of pores and BJH desorption average pore radius. Prior to analysis, the samples were placed in a measurement ampoule and degassed for 4 h at 300 °C.

#### 4.8.2. Powder X-ray Diffraction (XRD)

Room temperature powder X-ray diffraction patterns were recorded using a PANalytical X’Pert Pro MPD diffractometer in the Bragg-Brentano reflection geometry. Copper CuKα radiation was used from a sealed tube. Data were collected in the 2Θ range at 5°–90° with a step of 0.0167° and exposure per step of 27 s. The samples were spun during data acquisition to lower possible preferred orientation. APANalytical X’Celerator detector was used, based on Real Time Multiple Strip technology and capable of simultaneously measuring intensities in the 2Θ range of 2.122°. For qualitative analysis and to estimate crystallite sizes, PANalytical High Score Plus software was used, combined with powder diffraction file PDF-2 ver. 2009 from the International Centre for Diffraction Data (ICDD) database of standard reference materials. Based on the diffractograms obtained, the sizes of the palladium crystallites were estimated using the Scherrer equation (Table 11).

## 5. Conclusions

This study represents an initial step towards developing a system for the conversion of low-cost wastes from the sugar industry into valuable chemicals (furfural, furfuryl alcohol, tetrahydrofurfuryl alcohol, lactic acid, and propylene glycol), fuels (hydrogen and methane) or yeast biomass. The advantages of the solution presented here include not only the value of the end products, but also that it enables virtually “zero-waste” production. Currently, the sugar industry produces vast amounts of waste biomass, only part of which can be used without processing, as components in animal feed. Conversion of sugar beet leaves, around 2.5 × 10^9^ kg of which are produced each year in Poland alone, would be particularly profitable, since these leaves are currently left to rot once the sugar beets have been harvested, and then plowed. Our results show that FA and THFA can be obtained by catalytic reduction of furfural present in crude condensates. This is important because furfural separation and purification processes are costly and environmentally damaging, due to the use of organic solvents.

In the process described in this paper, acid hydrolysis of sugar beet pulp and leaves enables the simultaneous production of both furfural and a medium for microorganism cultivation. This medium can be used as a feedstock for single cell protein, which can be used to enrich the animal fodder already produced in sugar factories. The influence of the mode of preservation (freezing, ensiling and drying) and of storage time on the yield of furfural from the waste biomass was also investigated. All tested methods of fixation provided high yields of furfural, comparable with those from fresh material. Long-term storage of ensiled waste biomass did not result in loss of furfural productivity. This confirms the usefulness of these methods for the commercial production of furfural. Moreover, the destination residue was found to be a suitable feedstock for the production of microbiological media. Carbohydrates determined in the hydrolysates were utilized by both yeast strains and lactic acid bacteria. It is thus possible to obtain both single cell protein and lactic acid, making the concept of recycling waste biomass from the sugar industry more attractive.

## Figures and Tables

**Table 1:** Influence of preservation methods on the concentration and volume of furfural obtained in distillates from waste materials.

Biomass Sample	Furfural Concentration in Condensate (mmol/L)	Condensate Volume (mL)	Yield of Furfural (wt. %)	Dry Mass (%)
W	54.33 ± 1.71	238.0 ± 6.56	4.97 ± 0.29	21.12
W1	55.63 ± 2.01	217.7 ± 5.69	4.66 ± 0.25	19.34
WP	74.50 ± 3.14	167.3 ± 6.03	4.79 ± 0.03	92.90
WF	40.00 ± 1.98	301.0 ± 12.73	4.63 ± 0.04	20.02
WD	84.95 ± 2.48	112.1 ± 6.41	3.66 ± 0.13	100
L	16.67 ± 4.53	218.7 ± 38.44	1.41 ± 0.45	19.69
L1	15.37 ± 0.87	224.0 ± 6.56	1.32 ± 0.10	46.35
LD	29.80 ± 5.00	172.3 ± 11.68	1.96 ± 0.21	100.0
LF	34.53 ± 2.16	154.3 ± 11.15	2.04 ± 0.13	18.58

**Table 2:** Influence of ensilage and storage time on the concentration and volume of furfural obtained in distillates from waste materials.

Biomass Sample	Furfural Concentration in Condensate (mmol/L)	Condensate Volume (mL)	Yield of Furfural (wt. %)	Dry Mass (%)
W1	55.64 ± 2.00	217.7 ± 5.69	4.66 ± 0.25	19.34
W2	36.44 ± 1.64	311.7 ± 24.83	4.36 ± 0.27	18.27
W3	36.15 ± 4.74	328.0 ± 19.97	4.56 ± 0.68	14.81
W4	24.33 ± 8.29	292.0 ± 4.00	2.73 ± 0.95	16.51
W5	42.98 ± 6.03	324.3 ± 11.37	5.34 ± 0.58	17.03
L1	15.35 ± 0.88	224.0 ± 6.56	1.32 ± 0.10	46.35
L2	13.86 ± 0.75	230.0 ± 14.53	1.22 ± 0.01	45.05
L3	12.75 ± 3.20	239.7 ± 8.74	1.17 ± 0.26	44.65

**Table 3:** Conversion of furfural over Pd/Al_2_O_3_ catalysts and yields of FA and (THFA).

t (h)	T (°C)	pH_2_ (atm)	m_cat_ (g)	C_0_ (M)	X (%)	Y_FA_ (%)	Y_THFA_ (%)	Y_THF,_ Y_MTHF_ (%)
2	60	20	0.50	0.10	100	44.79	55.12	-
	90				100	27.71	70.96	1.20 _THF_
	120				100	5.69	59.53	unidentified products
2	90	10	0.50	0.10	86.0	34.49	42.56	0.71_THF_ + 0.15_MTHF_
		20			100	27.71	70.96	1.20_THF_
		35			100	0	96.56	0.55_MTHF_
2	90	20	0.25	0.10	79.9	51.71	27.75	0.37_THF_
			0.50		100	27.71	70.96	1.20_THF_
			0.75		100	29.12	69.87	0.60_THF_
1	90	20	0.5	0.10	92.74	48.69	47.36	-
2					100	27.71	70.96	1.20_THF_
4					100	5.92	93.70	0.80_THF_
2	90	20	0.5	0.10	100	27.71	70.96	1.20_THF_
				0.50	56.17	33.22	22.95	-
				1.00	34.19	16.06	0	-
				12.0	24.25	4.82	0	-

Activation of catalysts: drying in air at 110 °C, 6 h; reduction in H_2_ at 300 °C, 2 h.

**Table 4:** Carbohydrate profiles of obtained biomass hydrolysates.

	Compound (g/L)								
Medium	Glucose	Fructose	Mannose	Arabinose	Galactose	Raffinose	Rhamnose	Xylose	Galacturonic Acid
	**Sugar Beet Leaf Hydrolysates**								
L/A	0.22 ± 0.03	0.37 ± 0.04	0.37 ± 0.04	0.13 ± 0.01	0.28 ± 0.03	0.08 ± 0.01	1.02 ± 0.15	1.14 ± 0.01	0.37 ± 0.04
L/B	0.18 ± 0.03	2.08 ± 0.31	2.08 ± 0.27	2.97 ± 0.03	4.14 ± 0.45	0.00 ± 0.00	0.74 ± 0.07	0.69 ± 0.07	2.08 ± 0.24
L1/A	0.16 ± 0.02	1.21 ± 0.15	1.21 ± 0.01	3.52 ± 0.41	4.70 ± 0.05	0.07 ± 0.01	0.70 ± 0.06	1.73 ± 0.02	1.21 ± 0.01
L1/B	0.14 ± 0.02	1.45 ± 0.16	1.45 ± 0.02	2.66 ± 0.33	3.28 ± 0.04	0.10 ± 0.01	0.94 ± 0.10	0.85 ± 0.09	1.45 ± 0.01
L2/A	3.23 ± 0.36	0.77 ± 0.08	0.77 ± 0.06	0.00 ± 0.00	0.00 ± 0.00	8.88 ± 0.54	1.37 ± 0.21	1.38 ± 0.19	0.77 ± 0.08
L2/B	2.70 ± 0.16	0.29 ± 0.04	0.29 ± 0.03	0.00 ± 0.00	0.00 ± 0.00	6.04 ± 0.64	1.13 ± 0.12	0.74 ± 0.07	0.29 ± 0.03
L4/A	7.56 ± 0.85	0.55 ± 0.04	0.55 ± 0.04	0.00 ± 0.00	0.24 ± 0.03	4.22 ± 0.41	0.66 ± 0.07	0.85 ± 0.09	0.55 ± 0.06
L4/B	4.30 ± 0.36	0.54 ± 0.05	0.54 ± 0.05	0.00 ± 0.00	0.00 ± 0.00	5.27 ± 1.11	1.16 ± 0.21	1.82 ± 0.02	0.54 ± 0.06
	**Sugar Beet Leaf Hydrolysates**								
W/A	2.70 ± 0.33	0.08 ± 0.01	16.99 ± 1.55	12.12 ± 1.33	12.80 ± 1.36	6.04 ± 0.62	1.13 ± 0.17	3.13 ± 0.35	2.59 ± 0.29
W/B	7.56 ± 0.81	0.01 ± 0.01	15.16 ± 1.61	12.94 ± 1.41	14.10 ± 1.82	4.22 ± 0.35	0.66 ± 0.07	2.29 ± 0.28	0.76 ± 0.08
W1/A	3.83 ± 0.42	0.03 ± 0.01	16.08 ± 1.72	25.90 ± 2.66	26.76 ± 2.66	8.78 ± 0.91	1.33 ± 0.15	3.07 ± 0.32	4.18 ± 0.39
W1/B	4.30 ± 0.51	0.03 ± 0.01	16.70 ± 1.34	34.16 ± 3.52	38.60 ± 3.41	10.27 ± 1.37	1.16 ± 0.21	3.52 ± 0.41	3.34 ± 0.41
W2/A	0.16 ± 0.01	0.49 ± 0.05	0.13 ± 0.01	11.35 ± 1.45	14.52 ± 2.00	0.07 ± 0.01	0.70 ± 0.07	0.70 ± 0.07	0.25 ± 0.02
W2/B	0.18 ± 0.01	0.04 ± 0.01	0.19 ± 0.02	0.42 ± 0.05	0.71 ± 0.07	0.00 ± 0.00	0.74 ± 0.07	0.68 ± 0.07	0.27 ± 0.03
W4/A	0.14 ± 0.01	0.25 ± 0.03	0.21 ± 0.02	0.50 ± 0.05	0.83 ± 0.07	0.10 ± 0.01	0.94 ± 0.08	0.69 ± 0.07	0.21 ± 0.02
W4/B	0.07 ± 0.01	0.01 ± 0.00	0.22 ± 0.02	0.52 ± 0.06	0.73 ± 0.07	0.10 ± 0.01	0.77 ± 0.08	0.37 ± 0.03	0.33 ± 0.03

A, B—samples collected from different parts of biomass prisms.

**Table 5:** Growth of selected industrial yeast strains on biomass hydrolysates.

Strain	Stage	Sugar Beet Leaf Hydrolysate								Sugar Beet Pulp Hydrolysate							
		L/A	L/B	L1/A	L1/B	L2/A	L2/B	L4/A	L4/B	W/A	W/B	W1/A	W1/B	W2/A	W2/B	W4/A	W4/B
		Increase in optical density (Δ McF)															
*S. cerevisiae* Tokay	I	1.0	0.6	1.3	1.6	1.6	1.2	0.6	0.9	1.0	0.0	0.5	0.0	0.0	0.5	1.5	1.4
	II	0.0	0.5	0.1	0.0	0.0	0.1	0.0	0.0	1.2	0.7	2.7	0.5	0.2	0.1	1.4	0.1
*S. pastorianus* 203	I	0.9	1.0	1.8	2.0	1.8	1.5	1.5	2.9	1.4	1.7	4.4	1.7	0.5	0.1	1.0	0.0
	II	0.2	0.4	0.3	0.4	0.0	0.3	0.0	0.0	1.0	1.5	0.6	4.4	0.0	0.2	0.5	1.9
*S. cerevisiae* Etanol Red	I	2.0	1.5	1.7	1.5	1.5	1.8	1.4	1.7	0.7	1.9	4.0	0.6	0.2	0.2	0.4	0.5
	II	0.0	1.3	0.1	0.0	0.1	0.1	0.0	0.0	0.9	1.6	3.5	0.0	0.0	0.5	2.7	1.4
*S. cerevisiae* 1183	I	0.9	1.5	1.1	1.5	2.2	1.6	1.4	1.7	0.3	1.0	4.3	1.6	0.3	0.0	0.9	0.6
	II	0.2	0.6	0.2	0.5	0.5	0.1	0.1	0.1	0.0	0.1	3.8	0.4	0.0	0.0	1.7	0.5
*S. cerevisiae* TT	I	1.2	1.5	2.2	2.0	1.8	1.8	2.0	1.8	1.0	1.0	0.5	1.5	0.0	0.0	1.0	2.0
	II	0.0	0.9	0.0	0.0	0.0	0.2	0.9	0.7	0.9	0.4	4.0	2.0	0.1	0.0	1.2	0.1
*S. cerevisiae* Ja64	I	0.8	0.0	1.9	2.0	2.4	1.6	2.2	1.0	0.5	0.5	0.5	0.0	0.5	1.0	1.0	2.1
	II	0.0	0.4	0.1	0.0	0.3	0.0	0.4	0.7	0.0	1.0	3.8	0.4	0.1	0.5	0.2	0.6
*S. pastorianus* 1116	I	1.0	1.0	0.5	1.5	0.7	1.4	0.5	0.7	0.0	0.0	1.0	1.0	0.5	0.5	1.5	0.7
	II	0.1	0.1	0.2	0.2	0.0	0.0	0.1	0.0	0.5	1.6	3.0	4.5	0.0	0.2	0.4	1.8
*S. cerevisiae* V1116	I	1.3	1.5	1.0	1.5	2.5	1.7	1.5	2.0	1.3	0.5	0.0	0.0	0.0	0.5	0.0	1.0
	II	0.0	0.8	0.1	0.1	0.1	0.2	0.3	0.0	0.0	0.2	0.9	3.7	0.0	0.0	0.1	0.9

A, B—samples collected from different parts of biomass prisms.

**Table 6:** Growth of selected non-conventional yeast strains on biomass hydrolysates (increase in optical density (Δ McF)).

Strain	Stage	Sugar Beet Leaf Hydrolysate								Sugar Beet Pulp Hydrolysate							
		L/A	L/B	L1/A	L1/B	L2/A	L2/B	L4/A	L4/B	W/A	W/B	W1/A	W1/B	W2/A	W2/B	W4/A	W4/B
*P. angusta* 495	I	1.5	1.0	1.5	1.5	1.5	1.3	1.7	1.5	0.5	1.0	0.5	1.0	0.5	0.5	0.0	1.0
	II	0.1	0.2	0.3	0.3	0.1	0.3	0.3	0.3	1.0	2.0	4.1	0.7	0.4	0.5	0.6	0.0
*K. marxianus* 179	I	1.5	1.0	1.3	1.0	1.7	2.0	2.1	2.5	2.1	2.1	3.7	2.3	0.5	0.5	1.5	0.4
	II	0.3	2.2	0.4	0.1	0.3	0.4	0.6	0.0	0.8	0.0	1.3	3.2	0.0	0.3	0.9	1.3
*K. marxianus* 0028	I	0.6	1.0	1.7	1.5	2.0	1.2	1.8	1.2	1.6	1.3	1.8	0.0	1.1	1.0	1.7	1.1
	II	0.3	1.9	2.4	1.8	0.2	0.9	0.7	0.0	0.8	2.3	3.8	0.0	2.1	2.9	2.5	2.1
*C. utilis* 0021	I	0.3	1.8	1.5	1.0	1.5	1.8	0.7	1.0	3.7	2.9	2.8	3.4	3.7	2.8	2.9	2.0
	II	0.3	2.8	1.7	2.2	1.4	0.8	0.6	0.7	4.6	3.1	3.9	4.4	2.7	3.3	1.3	1.3

A, B—samples collected from different parts of biomass prisms.

**Table 7:** Growth of selected lactic acid bacteria strains on biomass hydrolysates (increase in optical density (Δ McF)).

Strain	Stage	Sugar Beet Leaf Hydrolysate								Sugar Beet Pulp Hydrolysate							
		L/A	L/B	L1/A	L1/B	L2/A	L2/B	L4/A	L4/B	W/A	W/B	W1/A	W1/B	W2/A	W2/B	W4/A	W4/B
*Lb. plantarum* AX-G	I	2.1	0.0	0.0	0.0	0.0	1.0	3.2	1.1	1.5	0.5	1.0	0.0	0.0	0.5	2.0	2.4
	II	0.2	0.4	1.3	1.8	0.3	0.5	1.7	0.0	2.0	3.2	2.0	2.0	0.1	0.0	1.0	0.4
*Lb. plantarum 8014*	I	2.1	0.0	1.0	0.0	1.6	0.4	0.8	1.4	2.2	2.0	2.3	2.1	0.8	0.3	0.3	0.5
	II	0.5	1.2	0.5	1.1	0.4	1.5	0.2	0.1	0.6	1.1	0.0	2.3	0.0	0.3	0.1	0.0
*Lb. plantarum* 2675	I	2.3	0.0	1.0	0.0	1.1	1.1	1.3	0.9	2.1	0.4	2.1	2.7	0.8	0.5	1.5	0.4
	II	0.0	2.3	0.3	0.6	0.0	0.9	0.0	0.0	0.6	0.0	3.1	0.2	0.4	0.2	1.1	1.4
*Lb. plantarum* AX-D	I	1.0	0.0	2.0	0.0	2.3	1.6	1.4	1.1	2.9	2.1	1.5	3.0	1.0	0.8	1.6	0.5
	II	0.9	1.6	0.3	0.1	0.6	0.0	0.0	0.0	1.4	0.1	1.5	0.5	0.1	0.1	0.6	0.8
*Lb. brevis* 488	I	1.5	1.0	1.0	0.0	0.9	0.5	1.3	1.2	4.1	4.4	4.3	4.7	2.8	1.9	1.0	1.6
	II	0.3	1.6	0.1	0.0	0.7	2.3	0.0	0.0	1.1	2.7	2.9	3.5	0.9	1.0	0.4	0.6

A, B—samples collected from different parts of biomass prisms.

**Table 8:** Specific surface area and textural properties of the studied supports.

Support	Surface Area (BET) (m^2^/g)	Average Pore Size (BJH) (nm)
Kaolin (Chinafil)	9.44	8.50
Kaolin (KOG)	9.61	8.40
Kaolin (GL)	29.3	5.21
Kaolin (BG)	33.2	4.70
SiO_2_	320	4.50
Al_2_O_3_	111	6.00
TiO_2_	57.0	15.0
C _AG5_	960	-

**Table 9:** Biomass used in the investigation.

Type of Sample	Preservation Methods	Date of Sampling *	Dry Mass (%) **	Symbol
Sugar beet pulp	Fresh	18 November 2016	21.12	W
	Frozen	24 November 2016	20.02	WF
	Palletized	2 December 2016	92.90	WP
	Dried	21 November 2016	100	WD
	Ensiled	6 December 2016	19.34	W1
		10 January 2017	18.27	W2
		21 February 2017	14.81	W3
		3 April 2017	16.51	W4
		8 May 2017	17.03	W5
Sugar beet leaves	Fresh	18 November 2016	19.69	L
	Frozen	25 November 2016	18.58	LF
	Dried	8 December 2016	100	LD
	Ensiled	6 December 2016	46.35	L1
		10 January 2017	45.05	L2
		21 February 2017	44.65	L3
		3 April 2017	48.34	L4

* A minimum of three samples of each plant material were taken. ** Average value calculated from measuring the dry weight of each sample.

**Table 10:** Summary of validation parameters for GC-FID analysis of F, FA and THFA.

Parameter	Analyte	Value
Working range	F	0.0005–0.014 mol/L
	FA	0.0005–0.014 mol/L
	THFA	0.0005–0.014 mol/L
Coefficient of determination R^2^	F	0.9993
	FA	0.9993
	THFA	0.9997
Limit of quantification	F	1.2 × 10^−4^ mol/L
	FA	1.2 × 10^−4^ mol/L
	THFA	2.8 × 10^−4^ mol/L
Trueness	F	9.81%
Coefficient of variation for repeatability conditions	F	4.14%
	FA	3.79%
	THFA	4.01%
Coefficient of variation for reproducibility conditions	F	7.22%
	FA	12.71%
	THFA	12.40%
Recovery	F	91.86%
	FA	101.75%
	THFA	92.55%

**Table 11:** Size of palladium crystallites estimated on the basis of XRD results using the Scherrer equation.

Catalyst	Size of Palladium Crystallites Estimated on the Basis of the Peak Pd (111) (nm)
5%Pd/Al_2_O_3_	7
5%Pd/SiO_2_	40
5%Pd/TiO_2_	10
5%Pd/ C_AG5_	cannot be estimated
5%Pd/Kaolin (KOG)	10
5%Pd/Kaolin (Chinafill)	8
5%Pd/Kaolin (GL)	9
